# Long-term effects of three Tiao-Bu Fei-Shen therapies on NF-κB/TGF-β1/smad2 signaling in rats with chronic obstructive pulmonary disease

**DOI:** 10.1186/1472-6882-14-140

**Published:** 2014-04-26

**Authors:** Ya Li, Jian-sheng Li, Wei-wei Li, Su-yun Li, Yan-ge Tian, Xiao-fan Lu, Su-li Jiang, Ying Wang

**Affiliations:** 1 Institute of Respiratory Disease, First Affiliated Hospital, Henan University of Traditional Chinese Medicine, Zhengzhou, Henan 450000, China; 2 Central Laboratory, First Affiliated Hospital, Henan University of Traditional Chinese Medicine, Zhengzhou, Henan 450000, China; 3 Institute of Gerontology, Henan University of Traditional Chinese Medicine, Longzihu University Park, Zhengzhou, Henan 450046, China

**Keywords:** Chronic obstructive pulmonary disease, Bufei jianpi, Bufei yishen, Yiqi zishen, Traditional Chinese medicine

## Abstract

**Background:**

The three Tiao-Bu Fei-Shen (Bufei Jianpi, Bufei Yishen, Yiqi Zishen) granules have been confirmed for their beneficial clinical efficacy in chronic obstructive pulmonary disease (COPD) patients on reducing frequency and duration of acute exacerbation, improving syndromes, pulmonary function and exercise capacity. But the short- or long-term mechanism of them is not fully clear. Nuclear factor (NF)-κB/transforming growth factor (TGF)-β1/smad2 signaling pathway is involved in the progress of inflammation and remodeling in chronic obstructive pulmonary disease COPD. This study aimed to explore the long-term effects mechanism of Tiao-Bu Fei-Shen granules by regulating NF-κB/TGF-β/Smads signaling in rats with COPD.

**Methods:**

Sprague Dawley rats were randomized into control, model, Bufei Jianpi, Bufei Yishen, Yiqi Zishen and aminophylline groups. COPD rats, induced by cigarette smoke and bacterial infections exposures, were administrated intragastricly by normal saline, Bufei Jianpi, Bufei Yishen, Yiqi Zishen granules or aminophylline from week 9 through 20, respectively. At week 20 and 32, lung tissues were harvested. Immunohistochemistry was used to detect interleukin (IL)-1β and tumor necrosis factor (TNF)-α, quantitative real-time polymerase chain reaction (qRT-PCR) was used for TGF-β1 and Smad2 mRNA analysis, western blotting was used to determine the phosphorylation of NF-κB (p-NF-κB) and IκBα (p-IκBα).

**Results:**

COPD rats had marked airway injury, such as chronic airway inflammation and remodeling, emphysema, which were improved in the three traditional Chinese medicines (TCM)-treated animals. The levels of IL-1β, TNF-α, p-NF-κB, p-IκBα, TGF-β1 and Smad2 were significantly higher in COPD rats than in controls, while they were dramatically reduced in the three TCM- and aminophylline-treated groups. At the meantime, all these endpoints were significantly lower in three TCM-treated groups than in aminophylline group, especially in Bufei Jianpi and Bufei Yishen groups. Compared to week 20, all endpoints decreased significantly in three TCM groups at week 32.

**Conclusion:**

The three Tiao-Bu Fei-Shen therapies can reduce pulmonary inflammation and remodeling in COPD and have significant long-term effects. NF-κB/TGF-β1/smad2 signaling might be involved in the mechanism.

## Background

Chronic obstructive pulmonary disease (COPD), a prevalent smoking-related disease for which no disease-altering therapies currently exist, is characterized by persistent airflow limitation and progressive pathology that resulted from recurrent inflammation and remodeling in small airway. These pathological damages occur throughout the course of COPD [1]. Nuclear factor (NF)-κB/transforming growth factor (TGF)-β1/Smads2 signaling pathway as an important pathway involved in inflammation and remodeling is closely related to the progress of COPD. NF-κB, a transcriptional activation protein, is widely involved in various biological processes, such as inflammation, oxidative stress, immune response, etc. The phosphorylation of NF-κB can initiate the gene transcriptions and expressions of various cytokines, amplify the inflammatory response through a positive feedback cascade. Additionally, NF-κB can also promote the expression of TGF-β1 and activate TGF-β1/Smad2 signaling by binding with NF-κB combining site locating in TGF-β1 activating factor-tissue transglutaminase (tTG) gene promoter [2]. TGF-β1, an important proinflammatory cytokine with strong fibrotic effect, plays a critical role in inflammatory injury and repair as well as airway remodeling in COPD by activating Smad2, a downstream receptor kinase of TGF-β1 [3,4]. The effects of NF-κB/TGF-β/Smads pathways are as follows: 1) to promote the secretion of inflammatory cytokines and chemokines, augment inflammatory response; 2) to induce fibroblast cell differentiation into highly synthetic myofibroblasts and arguably transdifferentiation of epithelial cells into fibroblasts, produce more cytokines to aggravate the lung injury; 3) to increase the production of collagen fibers, elastic fibers and reticular fibers, promote them deposit in the cell membrance, which may lead to airway wall thickening and ultimately aggravate airflow limitation; 4) to increase the secretion of the extracellular matrix components (such as bronchopulmonary tissue fibronectin, collagen type III and I glycoprotein, etc.) and finally result in airway plasticity decreasing and remodeling [3-6].

COPD belongs to the category of lung distension (FEIZHANG Disease) in traditional Chinese medicine (TCM). In previous investigations, we found that the patterns of lung-spleen qi (vital energy in life body) deficiency, lung-kidney qi deficiency and lung-kidney qi-yin (vital energy in life body and its material base, such as blood, flesh, bones, etc.) deficiency were the most common syndromes in the stable stage of COPD [7,8]. Accordingly, we formulated the three Tiao-Bu Fei-Shen (Bufei Jianpi/invigorating the lung and strengthening the spleen, Bufei Yishen/invigorating the lung and reinforcing the kidney, Yiqi Zishen/replenishing qi and nourishing the kidney) therapies and granules. In two previous randomized controlled trials, we found that the three Tiao-Bu Fei-Shen granules can alleviate clinical symptoms in stable COPD patients, reduce frequency and duration of acute exacerbation and improve pulmonary function, exercise capacity and stamina and quality of life [9]. In animal studies, it indicates that the three TCM granules can reduce pulmonary and systemic inflammation, decrease collagen deposition and metalloproteinase expression, reduce pathological impairment and improve pulmonary function and have marked long-term effects [10-12]. In this article, we aimed to explore the long-term effects of the three Tiao-Bu Fei-Shen granules on airway inflammation and remodeling by regulating NF-κB/TGF-β1/Smads2 signaling in COPD rats.

## Methods

### Animals

Sixty male and sixty female two-month-old Sprague–Dawley rats were purchased from Experimental Animal Center of Henan province (Zhengzhou, China), weighing 180–220 g. All rats arrived at the animal facility of the laboratory seven days before the experiment were housed in the individually ventilated cages (CA25, Fengshi, Suzhou, China) and provided free access to sterile food and water.

### Klebsiella pneumoniae

*Klebsiella pneumoniae* (strain ID: 46114) purchased from National Center For Medical Culture Collection (CMCC, Bejing, China) was cultured and prepared into a suspension of 6 × 10^8^ colony forming units (CFU) per milliliter (mL) with normal saline before administered to animals.

### Preparation of COPD model

Experimental protocol was approved by the Experimental Animal Care and Ethics committees in the First Affiliated Hospital, Henan University of Traditional Chinese Medicine, Zhengzhou, China.

Rats were randomized into control, model, Bufei Jianpi, Bufei Yishen, Yiqi Zishen and aminophylline groups (10 male and 10 female rats in each group). COPD Rats (except control ones) were exposed to tobacco (Hongqi Canal® Filter tip cigarette, tobacco type, tar: 10 mg, nicotine content: 1.0 mg, carbon monoxide: 12 mg, Henan Tobacco Industry, Zhengzhou, China) smoke of 8 cigarettes per treatment, twice a day, during the first two weeks; then fifteen cigarettes per treatment, three times a day, from week 3 through week 12. The rats were placed in a sealed box connected to smoke source to receive two or three 30-minute exposures per day, with one or two three-hour intervals. One hundred μL of *Klebsiella pneumonia* suspension (6 × 10^8^ CFU/mL) was slowly dropped into nasal cavities in cigarette smoke-exposed rats, per 5 days, from week 1 through 8 [13-17].

### Administrations

At the end of week 8, two COPD rats were sacrificed to validate whether the model were successfully made or not according to the pathological changes of lung tissue and pulmonary function impairment. The rest rats were intragastrically administrated by normal saline (2 mL per animal, in control and model rats), Bufei Jianpi granule (4.84 g/kg/d, in Bufei Jianpi group), Bufei Yishen granule (4.44 g/kg/d, in Bufei Yishen group), Yiqi Zishen granule (4.84 g/kg/d, in Yiqi Zishen group) and aminophylline (2.3 mg/kg/d, in aminophylline group) from week 9 through 20, respectively. Animal necropsies were executed at week 20 and 32.

The main components of the granules are as follows: Bufei Jianpi granules (For lung-spleen qi deficiency syndrome): Huang Qi (*Astragalus propinquus*) 15 g, Dang Shen (*Codonopsis pilosula*) 15 g, Bai Zhu (*Atractylodes macrocephala*) 12 g, Fu Ling (*Poria cocos*) 12 g; Bufei Yishen granules (For lung-kidney qi deficiency syndrome): Ren Shen (*Radix ginseng*) 9 g, Huang Qi (*Astragalus propinquus*) 15 g, Shan Zhu Yu (*Cornus officinalis*) 12 g and Yin Yang Huo (*Herba epimedii*) 9 g; Yiqi Zishen granule (For lung-kidney qi and yin deficiency syndrome): Ren Shen (*Radix ginseng*) 9 g, Huang Jing (*Polygonatum kingianum*) 15 g, Shu Di Huang (*Radix rehmanniae* praeparata) 15 g, Mai Dong (*Ophiopogon japonicus*) 15 g and Wu Wei Zi (*Schisandra chinensis*) 9 g. The herbs were purchased from market and identified and prepared into fluidextractum according to the standard operating procedure by the Department of Pharmaceutics in the First Affiliated Hospital, Henan University of Traditional Chinese Medicine, Zhengzhou, China. The human equivalent doses of the three TCM prescriptions and aminophylline were calculated by the following formula according references [9,10]. D_rat_ = D_human_ × (K_rat_/K_human_) × (W_rat_/W_human_)^2/3^[18]. D: dose (mg/Kg); K: body shape index; W: body weight.

### Morphology

At week 20 and 32, lung tissues were sampled after euthanasia and cut into 3-millimeter thick slices along the maximum diameter of the right lower lobe and fixed in 4% paraformaldehyde for 72 hours. The samples were embedded with paraffin, 4 μm thick sections were cut and stained with hematoxylin and eosin (H&E). All images were taken at amplification of 200 under an Olympus PM-10 AD optical microscope and photographic system (Olympus, Tokyo, Japan). Bronchia and lung alveolar were observed under optical microscope and bronchial wall thickness were measured by Image-Pro Plus® (IPP) 6.0 software (Media Cybernetics, MD, USA). Four bronchial wall thickness were measured in each slice and averaged.

### Immunohistochemistry

The expressions of IL-1β and TNF-α in lung tissues were analyzed by immunohistochemistry.

Paraffin-embedded lung tissue was sliced into 4 μm thick slices, deparaffinized and blocked with 3% hydrogen peroxide solution for 10 minutes to eliminate the activity of endogenous peroxidase and then incubated in polyclonal anti-IL-1β and TNF-α antibody solution (1:100 dilution) overnight at 4°C, respectively. Subsequently washed with phosphate buffer solution (PBS) three times, the slices were incubated with horseradish peroxidase (HRP)-labeled anti-mouse immunoglobulin G (IgG) and counterstained with hematoxylin. In each section, five random fields were photographed at a magnification of 400×. Semiquantitative evaluations were performed by using IPP 6.0 software.

### Quantitative real-time PCR analysis

The expressions of TGF-β1 and smad2 mRNAs of lung tissues were analyzed by quantitative real-time PCR (qRT-PCR).

Total RNA was extracted by using TRIzol reagent (Life Technologies, NY, USA) according to the instruction and assessed by agarose gel electrophoresis and absorbance measurements at 260 and 280 nanometer (nm) wavelength on SP-1901 ultraviolet light spectrophotometer (Jinpeng Analytic Instrument Company, Shanghai, China). Reverse transcription (RT) was proceeded by using Supre® III First-Strand Synthesis Super Mix for qRT-PCR Kit (Life Technologies, NY, USA), while PCR was performed by using Platinum SYBR® Green® Super Mix-UDG Kit (Life Technologies, NY, USA). The reaction systems was prepared following the instructions of the kits. The initial activation was at 95°C for 15 s, 60°C for 15 s and 72°C for 30 s on an ABI 7300 real time instrument (ABI, CA, USA).

The primers of TGF-β1 and smad2 were designed and synthesized by Generay Biotech Co. Ltd. (Shanghai, China) and sequences are as follows: TGF-β1, forward (5′-3′): ATA GCA ACA ATT CCT GGC GTT ACC, reverse (5′-3′): CAC TGA AGC GAA AGC CCT GTA TTT; Smad2, forward (5′-3′): GCC GAG TGC CTA AGT GAT CAG, reverse (5′-3′): TTA ACA GA C TGA GCC AGA AGA GAG; glyceraldehyde-3-phosphate dehydrogenase (GAPDH), forward (5′-3′): ACA GCA ACA GGG TGG TGG AC, reverse (5′-3′): TTT GAG GGT GCA GCG AAC TT.

### Western blotting analysis

Lung tissues were homogenized in the cold homogenization buffer, containing 100 mM Tris, 150 mM NaCl, 1% Triton-X, 0.1% sodium dodecy1 sulfate (SDS), 1 mmol/L ethylene diamine tetraacetic acid (EDTA), 1 mmol/L ethylene glycol tetraacetic acid (EGTA), 1 mmol/L phenylmethy1 sulfonylfluoride (PMSF), protease and phosphatase inhibitors, then centrifuged at 4°C at 12000 g for 1 h. The concentrations of total protein in the supernatant were detected by Bradford method and then 2% SDS and 5% 2-mercaptoethanol added before protein denaturalization at 95°C for 5 min. Fifty micrograms of protein was separated by sodium dodecyl sulfate-polyacrylamide gel electrophoresis (SDS-PAGE) and transferred to polyvinylidene difluoride (PVDF) membranes (Millipore, Bedfore, MA, USA). The membranes were blocked with 2% BSA in Tris-buffered saline containing 20 mmol/L Tris-buffered saline (pH 7.4), 500 mmol/L NaCl and 0.05% Tween 20, then incubated with primary antibody, phosphorylation of nuclear factor -κB (p-NF-κB), inhibitor of κBα (p-IκBα) or β-actin (Santa Cruz, CA, USA) according to its instructions and horseradish peroxidase (HRP)-conjugated secondary antibodies (Santa Cruz, CA, USA). Finally, signals were gained by using the Super ECL (enhanced chemi-luminescence) Plus reagent (Solarbio, Shanghai, China) and scanned and quantified by PowerLook 2100XL-USB scanner (UMAX, Taiwan) and Image J software (NIH, MD, USA).

### Statistical analysis

Values are expressed as mean ± standard deviation (SD). Statistical differences between groups were performed by One-Way ANOVA with SPSS 19.0 software package (SPSS, Chicago, IL, USA). Paired *T* test were used to detect the difference between week 32 and 20 in each group. The significance level is *P* < 0.05.

## Results

### Mortality

During the 32-week-period, two rats died in the model, Bufei Jianpi, Bufei Yishen, Yiqi Zishen and aminophylline groups respectively due to pulmonary abscess. One control rat died due to foot abscess.

### Histomorphology

**Figure 1:**
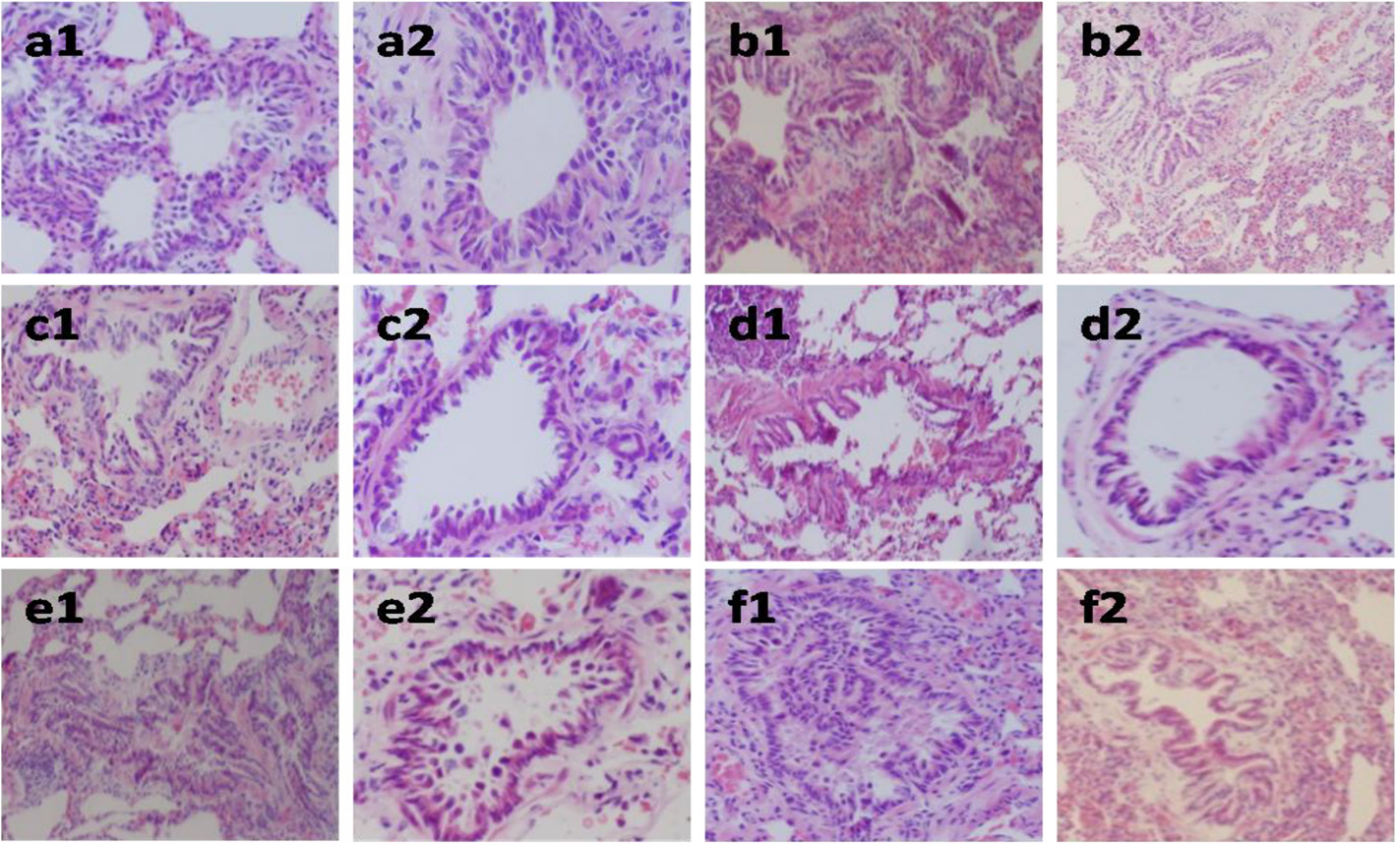
**Pathological changes in the lungs and bronchiole in rats treated with Bufei Jianpi, Bufei Yishen, Yiqi Zishen granules or aminophylline at week 20 and 32 ( H&E stained).** a = control, b = model; c = Bufei Jianpi; d= Bufei Yishen; e = Yiqi Zishen; f = aminophylline. 1 = week 20; 2 = week 32. Amplification ×200.

As shown in Figure 1, marked bronchiole stenosis, alveolar destruction, pulmonary bronchiole and arteriola wall thickening were observed in COPD rats at week 20 and 32, whereas they were reduced in the three TCM- and aminophylline-treated groups. No apparente impairment was observed in control rats.

### Bronchiole wall thickness

**Figure 2:**
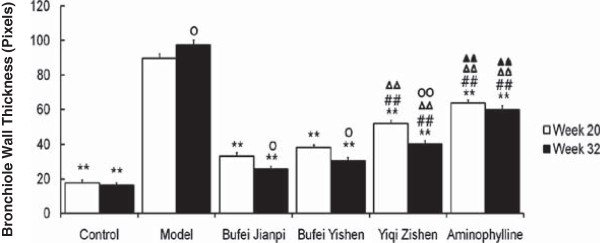
Bronchiole wall thickness in rats treated with Bufei Jianpi, Bufei Yishen, Yiqi Zishen granules or aminophylline at week 20 and 32. ^**^P<0.01, vs model group; ^##^*P*<0.01, vs Bufei Jianpi group; ^ΔΔ^*P*<0.01, vs Bufei Yishen group; ^▲▲^*P*<0.01, vs Yiqi Zishen group; ^○^*P*<0.05, ^○○^*P*<0.01, vs Week 20 in the same group.

As shown in Figure 2, bronchiole wall thickness in the model rats was 4-fold higher than that in control at both week 20 and 32 (*P* < 0.01). It was significantly lower in the three TCM- and aminophylline-treated group than in model rats (*P* < 0.01), while the three TCM-treated groups were significantly lower than aminophylline group (*P* < 0.01), especially in Bu-Fei Jian-Pi and Bu-Fei Yi-Shen groups (*P* < 0.01).

At week 32, bronchiole thickness in model group were higher compared with that at week 20 (*P* < 0.05), while the three TCM-treated groups were lower than those at week 20 (*P* < 0.05, *P* < 0.01).

### Cytokines

**Figure 3:**
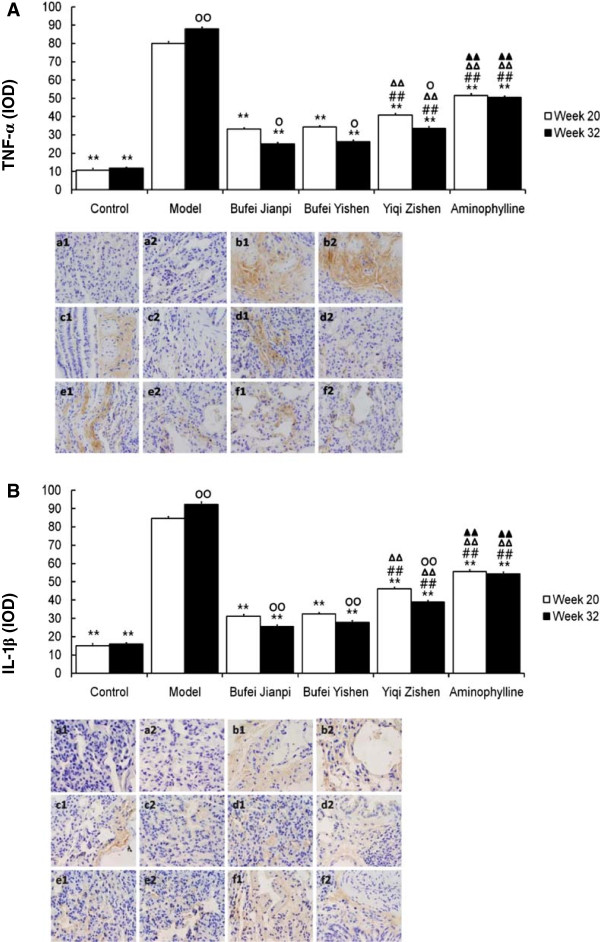
**Levels of TNF-α (A) and IL-1β (B) in the lungs in rats treated with Bufei Jianpi, Bufei Yishen, Yiqi Zishen granules or aminophylline at week 20 and 32.** a = control, b = model; c = Bufei Jianpi; d= Bufei Yishen; e = Yiqi Zishen; f = aminophylline. ^**^P<0.01, vs model group; ^##^*P*<0.01, vs Bufei Jianpi group; ^ΔΔ^*P*<0.01, vs Bufei Yishen group; ^▲▲^*P*<0.01, vs Yiqi Zishen group; ^○^*P*<0.05, ^○○^*P*<0.01, vs Week 20 in the same group.

As shown in Figure 3, the levels of TNF-α and IL-1β in the model group were significantly higher than those in control group at week 20 and 32 (*P* < 0.01). At the meanwhile, they were much lower in the three TCM- and aminophylline-treated group groups (all *P* < 0.01). The three TCM-treatments showed much more benefit than aminophylline (all *P* < 0.01), especially Bufei Jianpi and Bufei Yishen granules.

At week 32, the expressions of TNF-α and IL-1β were higher in model group than that at week 20 (*P* < 0.01), while they were significantly lower in the three TCM-treated groups (IL-1β, *P* < 0.05; TNF-α, *P* < 0.01).

### Phosphorylation of nuclear factor κB and inhibitor of κBα

**Figure 4:**
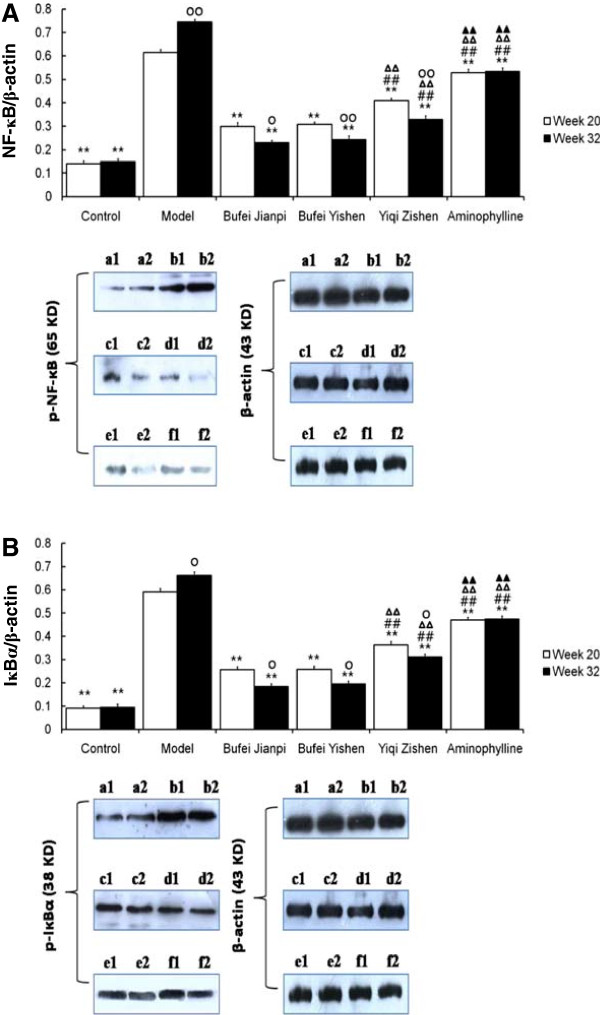
**Phosphorylation of NF-κB and IκBα of lung in rats treated with Bufei Jianpi, Bufei Yishen, Yiqi Zishen granules or aminophylline at week 20 and 32.** a = control, b = model; c = Bufei Jianpi; d= Bufei Yishen; e = Yiqi Zishen; f = aminophylline. ^**^P<0.01, vs model group; ^##^*P*<0.01, vs Bufei Jianpi group; ^ΔΔ^*P*<0.01, vs Bufei Yishen group; ^▲▲^*P*<0.01, vs Yiqi Zishen group; ^○^*P*<0.05, ^○○^*P*<0.01, vs Week 20 in the same group.

At week 20 and 32, the p-NF-κB and p-IκBα were significantly higher in model group than in control at both week 20 and 32 (*P* < 0.01) and the three TCM- and aminophylline-treated groups (*P* < 0.01) (Figure 4). In addition, the expressions of the proteins were lower in the three TCM-treated groups than aminophylline group (*P* < 0.01), especially in Bufei Jianpi and Bufei Yishen groups (*P* < 0.01).

At week 32, the phosphorylation levels of NF-κB and IκBα were higher in model group than week 20 (*P* < 0.01 and *P* < 0.05, respectively), while they were significantly lower in the three TCM-treated groups (*P* < 0.05 or *P* < 0.01).

### Expressions of TGF-β1 and Smad2 mRNA

**Figure 5:**
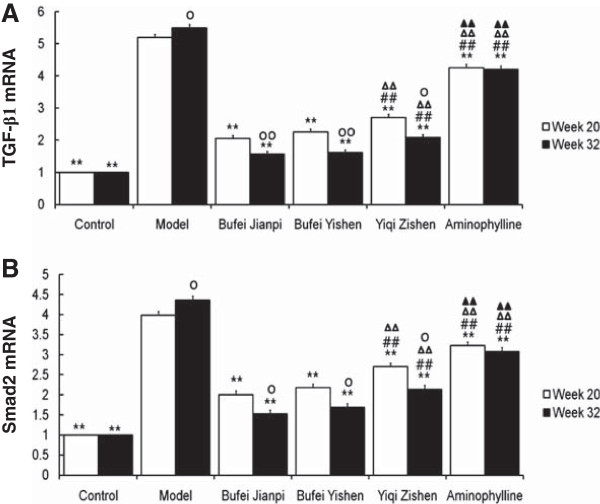
Expressions of TGF-β1 (A) and Smad2 (B) mRNA in rats treated with Bufei Jianpi, Bufei Yishen, Yiqi Zishen granules or aminophylline at week 20 and 32. ^**^P<0.01, vs model group; ^##^*P*<0.01, vs Bufei Jianpi group; ^ΔΔ^*P*<0.01, vs Bufei Yishen group; ^▲▲^*P*<0.01, vs Yiqi Zishen group; ^○^*P*<0.05, ^○○^*P*<0.01, vs Week 20 in the same group.

At week 20 and 32, the expressions of TGF-β1 and Smad2 mRNA were significantly higher in model group than in control (*P* < 0.01) and the three TCM- and aminophylline-treated groups (*P* < 0.01) (Figure 5). The expressions of mRNAs were significantly lower in three TCM-treated groups than aminophylline group (*P* < 0.01), especially in Bufei Jianpi and Bufei Yishen groups (*P* < 0.01).

At week 32, the expressions of TGF-β1 and Smad2 mRNA were higher in model group compared to that at -week 20 (*P* < 0.05, *P* < 0.01), conversely, they were significantly lower in three TCM-treated groups (*P* < 0.01, *P* < 0.05).

## Discussion and conclusions

The present study demonstrates that the three Tiao-Bu Fei-Shen granules play distinctly suppressant roles on inflammatory response and airway remodeling in COPD rats induced by cigarette smoke exposures combined with repeated bacterial infections via regulating NF-κB/TGF-β1/Smad2 pathway.

The syndromes of lung-spleen qi deficiency, lung-kidney qi deficiency and lung-kidney qi-yin deficiency are the most common syndromes in stable COPD. Bufei Jianpi, Bufei Yishen and Yiqi Zishen therapies and granules are the corresponding rules and medicines of treatments [7,8]. Previously completed multicenter clinical trials had shown that Bufei Jianpi, Bufei Yishen and Yiqi Zishen granules can improve pulmonary function, reduce the incidence and duration of acute exacerbation, improve patients’ quality of life, have beneficial long-term effects after a 12-month follow-up [9]. Therefore, this study aimed to evaluate the long-term effects of Bufei Jianpi, Bufei Yishen and Yiqi Zishen granules on pulmonary inflammation, airway remodeling and explore the role of NF-κB/TGF-β1/Smad2 signaling pathway on COPD rats.

Airway remodeling and airflow obstruction, resulted from chronic inflammation occurring in the central and peripheral airways as well as lung parenchyma, play important roles in the development of COPD [1,19,20]. NF-κB and TGF-β/Smads signaling pathways are the most important two paths involved in the course of inflammation and remodeling. NF-κB, a pivotal transcription factor of the inflammatory response, can be activated by numerous stimuli and participate in the regulation of hundreds of genes by binding to discrete DNA sequences, known as κB elements, in gene promoters and enhancers, such as TGF-β, TNF-α, IL-1β, etc. [21,22]. The expressions of NF-κB in plasma and sputum in COPD patients and smokers with normal lung function increase significantly compare with normal control subjects. IL-6, a downstream cytokine of NF-κB pathways, increased along with NF-κB [23]. Decreasing of phosphorylation of NF-κB can decrease the levels of downstream cytokines, reduce the inflammatory response in lungs of COPD, improve pulmonary function and finally reduce the incidences of acute exacerbation [21].

TGF-β1, a multipotential cytokine with strong fibrogenic effect, is presented in many tissues and cells in human body and is the most abundant isoform in the superfamily of transforming growth factors and with both structural and inflammatory cells being sources of TGF-β1 in the lungs [3]. In addition, TGF-β receptors are suggested to play a significant role in the pathogenesis of COPD through their regulation of TGF-β/Smads pathways. A previous study suggests that pharmacologic inhibition of TGF-β signaling can protect the murine lung from altered lung histology, impaired lung function and a panel of injury measures that accompany cigarette smoke-induced COPD [5].

There are strong interactions between NF-κB and TGF-β/Smads signaling to form NF-κB/TGF-β/Smads pathway as TGF-β can be administrated by NF-κB signaling pathway. NF-κB/TGF-β/Smads pathway leads on a global stage in airway inflammation and remodeling in COPD.

In this study, we have found that phosphorylation levels of NF-κB and IκBα, mRNA expressions of TGF-β1 and Smad2 increased significantly in the lungs of COPD rats. Bufei Jianpi, Bufei Yishen and Yiqi Zishen granules can reduce the phosphorylation of NF-κB and IκBα proteins and depress TGF-β1 and Smad2 mRNA expressions at both week 20 and 32. It indicates that the three Tiao-Bu Fei-Shen granules have a dramatic short- and long-term effect on reducing pulmonary pathological impairment and inflammatory responses, improving airway remodeling via regulating NF-κB/TGF-β1/Smad2 signaling. However, as the three traditional Chinese medicine prescriptions are very complicated compounds, it need more further study to determine what the exactly constituents make it work.

## Competing interests

The authors declare that they have no competing interests and they don’t receive any funding by Pharmaceutical factory.

## Authors’ contributions

LY, LJS and LSY contributed to the study design. LY also contributed to data analysis, manuscript drafting and process control. LSY also contributed process control and manuscript drafting. LWW contributed to protein analysis and data collection. TYG and LXF contributed to RNA analysis and data collection. JSL and WY contributed to animal experiment and cytokines analysis. All authors had read and approved the final manuscripts.

## Pre-publication history

The pre-publication history for this paper can be accessed here:

http://www.biomedcentral.com/1472-6882/14/140/prepub
